# Determination of Pesticide Residues in Vine Leaves Using the QuEChERS Method and Liquid Chromatography-Tandem Mass Spectrometry

**DOI:** 10.3390/foods13060909

**Published:** 2024-03-17

**Authors:** Mehmet Keklik, Ozgur Golge, Miguel Ángel González-Curbelo, Bulent Kabak

**Affiliations:** 1 Air Alaşehir Food Control Laboratory, Alaşehir 45600, Turkey; mehmet.keklik@hotmail.com; 2 Department of Gastronomy and Culinary Arts, Faulty of Tourism, Alanya Alaaddin Keykubat University, Alanya 07425, Turkey; ozgurgolge@gmail.com; 3 Departamento de Ciencias Básicas, Facultad de Ingeniería, Universidad EAN, Calle 79 no 11-45, Bogotá 110221, Colombia; magonzalez@universidadean.edu.co; 4 Department of Food Engineering, Faculty of Engineering, Hitit University, Corum 19030, Turkey; 5 Biotechnology Laboratory, Machinery and Manufacturing Technology Application and Research Center, Hitit University, Corum 19030, Turkey

**Keywords:** food safety, green leaves, LC-MS/MS, method validation, sample preparation, pesticides

## Abstract

Commercial viticulture necessitates regular pesticide applications to manage diseases and pests, raising significant concerns regarding pesticide residues among stakeholders. Due to health risks associated with these residues in Turkish vine leaves, the European Commission has increased the frequency of official control from 20% to 50%. Thus, the aim of this study was to determine multi-class pesticide residues in brined vine leaves from Turkey. A total of 766 samples of vine leaves were collected between May 2022 and June 2023. More than 500 residues were analyzed using the quick, easy, cheap, effective, rugged, and safe (QuEChERS) method, followed by liquid chromatography-tandem mass spectrometry. In-house validation data demonstrated that the analytical method exhibits fit-for-purpose performance in terms of linearity, accuracy, precision, and measurement uncertainty. Out of 766 samples analyzed, 180 samples (23.5%) contained one (131, 17.1%) or multiple (49, 6.4%) pesticides. Both the frequencies of occurrence and the rate of maximum residue level (MRL) exceedance increased in 2023 compared to 2022, with the MRL exceedance rate rising from 9.5% to 25.2%. Forty-three different residues were found in quantifiable concentrations and eight of them were non-approved. Among the residues, the non-systemic pyrethroid insecticides, lambda-cyhalothrin (8.0%) and cypermethrin (7.2%), were the two most frequently detected, with concentrations ranging from 0.010 to 0.248 mg kg^−1^ and from 0.011 to 0.533 mg kg^−1^, respectively. Turkey is a major exporter of vine leaves and these results provide crucial information regarding pesticide occurrence and quality assessment of vine leaves. The significant increase in both pesticide occurrence and MRL exceedance rates between 2022 and 2023 underscores the urgency for regulatory bodies to reassess current pesticide usage and monitoring practices. The findings emphasize the importance of implementing more stringent rules and improving enforcement methods in order to reduce the spread of unapproved pesticides and ensure adherence to global food safety standards.

## 1. Introduction

Viticulture stands as one of the most significant horticultural sectors globally, encompassing approximately 7 million ha of cultivated grapevines. The Mediterranean region serves as the primary hub for grapevine cultivation. Spain leads the world in cultivated area, boasting nearly 1 million ha, followed by France (0.76 million ha), Italy (0.70 million ha), China (0.58 million ha), and Turkey (0.39 million ha) in 2022 [1]. Various vine products, including table grapes, dried vine fruits (raisins, sultanas), wine, vinegar, alcoholic beverages (rakı, ouzo, tsipouro, arrack), grape juice, grape molasses, and vine leaves are regarded as valuable agricultural commodities for both domestic and international trade [2].

Vine leaves (*Vitis vinifera* L.), which are crucial for the plant’s growth and photosynthesis, have historically served various purposes, including food, medicine, and other uses. Primarily utilized in culinary contexts, vine leaves feature prominently in traditional gastronomic practices of the Mediterranean, Balkan, and Middle Eastern countries, including Turkey, Greece, Bulgaria, Lebanon, Syria, Israel, Cyprus, Iraq, and Iran. They are integral to the classic Turkish dish called dolma or sarma, which is produced by stuffing young vine leaves with a mixture of rice, onions, and various species and then cooked in olive oil [3].

Vine leaves serve as a rich source of dietary fiber; minerals (calcium, iron, magnesium, manganese, and phosphorus); and vitamins A, C, and K; as well as some phytochemicals [4]. Notably, leaves contain significant amounts of polyphenols (flavonoids and phenolics, such as hyperoside, catechin, astragalin, and quercetin), tocopherols (α- and γ-tocopherol), carotenoids (lutein, β-carotene), and phytosterols (β-sitosterol), all of which contribute to their nutritional and health benefits. The concentrations of these compounds can vary significantly depending on leaf variety [5]. Grapevine leaves offer benefits for numerous long-term health issues [6,7,8], in addition to exhibiting antimicrobial [9,10] and antioxidant properties [11,12,13].

The vine leaves are susceptible to infection by various fungal pathogens, including powdery mildew (*Erysiphe necator*), downy mildew (*Plasmopara viticola*) [14,15], gray mold (*Botrytis cinerea*), and *Phomopsis* cane and leaf spot (*Phomopsis viticola*) [16,17]. Moreover, viral infections, such as grapevine leafroll and grapevine fanleaf viruses, as well as phytoplasma diseases like flavescence dorée and Bois Noir, contribute to the spectrum of risks. The roster of adversaries extends to insect pests like thrips (*Anaphothrips vitis*, *Drepanothrips reuteri*, *Haplothrips globicies*), aphids (*Aphis illinoisensis*), aphid-like insects (phylloxera, *Daktulosphaira viti foliae*), grapevine moths (*Lobesia botrana*), mites (*Colomerus vitis*), and other leafhoppers (*Empoasca decipiens*, *Arboridia adanae*) [18,19].

Pesticides are the primary strategy for preventing the deterioration of vine crops and managing pests and plant infections in vineyards. A wide range of pesticides is typically applied to vineyards throughout different phases of cultivation and post-harvest storage. In Turkey, fungicides, such as azoxystrobin, boscalid, cyprodinil, and metalaxyl, and insecticides/acaricides, such as chlorpyrifos, are among the most commonly used pesticides in vineyards [20]. Contact or systemic fungicides have been reported to be widely used during the pre-harvest of vine leaves from Turkey, particularly against powdery mildew and downy mildew [21]. Although pesticides increase agricultural output, they can have both acute and long-term detrimental health consequences, as well as harmful effects on many terrestrial and aquatic non-target organisms when used excessively [22]. Therefore, pesticide use in agricultural production, as well as monitoring pesticide levels in fruits and vegetables, must be controlled and regulated to protect public health. Maximum residue limits (MRLs) for pesticides in agricultural products are determined by national and international agencies to establish food safety standards and promote international trade. In accordance with the European Union (EU) Regulation (EC) No. 396/2005 [23], the Turkish Ministry of Agriculture and Forestry establishes MRLs [24] for pesticide residues in both animal and plant products.

Numerous studies have demonstrated that vine leaves from Turkey contain substantial concentrations of different classes of pesticides [25,26,27,28]. These studies are based on only one-year data, which means upward or downward pesticide use trends cannot be identified. Surveillance of pesticide residues for over a year provides a more reliable and thorough dataset. On 27 October 2021, the European Commission published a regulation increasing the frequency of official control to be performed on vine leaves from Turkey from 20% to 50% due to the emergence of health risks from pesticide residues in vine leaves imported from Turkey [29].

Many extraction techniques have been developed for the extraction of pesticide residues from diverse food products in the last three decades. Some of the methods are liquid–liquid extraction [30], liquid-phase microextraction [31], solid-phase extraction [32], solid-phase microextraction [33], accelerated solvent extraction [34], supercritical fluid extraction [35], matrix solid-phase dispersion [36], and microwave-assisted extraction [37]. However, the main disadvantage of most of these technologies is that they are time consuming, labor intensive, sophisticated, expensive, and produce substantial waste. Alternatively, the quick, easy, cheap, effective, rugged, and safe (QuEChERS) method was developed by Anastassiades et al. [38] to improve the efficiency of traditional methods. The QuEChERS method has gained popularity over the last two decades due to its simplicity, speed, low cost, high throughput, and minimal solvent requirement. Contrary to the above advantages, the highly polar residues are not efficiently recovered from the matrices.

Thus, the primary objective of the current study was to assess the occurrence and concentration of multi-class residues in Turkish brined vine leaves over the course of two consecutive years (2022–2023). To achieve this, the QuEChERS method was employed in conjunction with liquid chromatography-tandem mass spectrometry (LC-MS/MS).

## 2. Materials and Methods

### 2.1. Reagents, Chemicals, and Standards

LC-gradient-grade methanol (MeOH), Lichrosolv^®^, was supplied from Supelco (Merck KGaA, Darmstadt, Germany) while HPLC-grade acetonitrile (ACN) and water were obtained from PanReac AppliChem (ITW Reagents, Darmstadt, Germany). Glacial acetic acid (CH_3_COOH, reagent grade, 100%) was purchased from Isolab (Wertheim, Germany). Analytical-grade ammonium formate (HCOONH_4_, analytical grade, ≥99% purity) and formic acid (CH_2_O_2_, LC-MS grade, 98–100% purity) were acquired from Sigma-Aldrich (Merck KGaA, Darmstadt, Germany). Both the QuEChERS kits (ChromaScience, İstanbul, Turkey) the salt extraction kit (AOAC 2007.01, containing 6 g of magnesium sulfate (MgSO_4_) and 1.5 g of sodium acetate (CH_3_COONa)), and the dispersive solid-phase extraction (d-SPE) kit (containing 1200 mg of MgSO_4_ and 400 mg of primary secondary amine (PSA)), were supplied from ChromaScience (İstanbul, Turkey).

Individual high purity (>95%) pesticide standards (*n* = 512) from a wide variety of chemical families were purchased from Dr. Ehrenstorfer GmbH (Augsburg, Germany) and Sigma-Aldrich (Steinheim, Germany). The selected pesticides included both the main pesticides used in vineyards in Turkey and other active chemicals that were either registered, non-registered, or banned. Each individual stock standard solution was prepared in ACN to a concentration of 1000 μg mL^−1^ and stored at −18 °C for a maximum of six months. From these individual solutions, a pesticide mix solution was prepared at a concentration of 10 μg mL^−1^ for each compound. Mixed standard working solutions at various concentrations were prepared by diluting the multi-analyte intermediate standard solution. These solutions were then used to create matrix-matched calibration standards and conduct method recovery tests.

### 2.2. Samples

Between May 2022 and June 2023, a total of 766 samples of brined vine leaves, belonging to the *Vitis vinifera* L. cultivar *Narince,* were collected from 155 different producers in the primary production areas of Tokat province, Turkey, for monitoring 512 multi-class pesticide residues. Each sample had a minimum weight of 1 kg. Sub-samples of vine leaves were homogenized using a home food processor (Sefa Çelik, Beyoğlu, İstanbul, Turkey) and stored under cool conditions (4–8 °C) until analysis.

### 2.3. Sample Preparation

**Figure 1:**
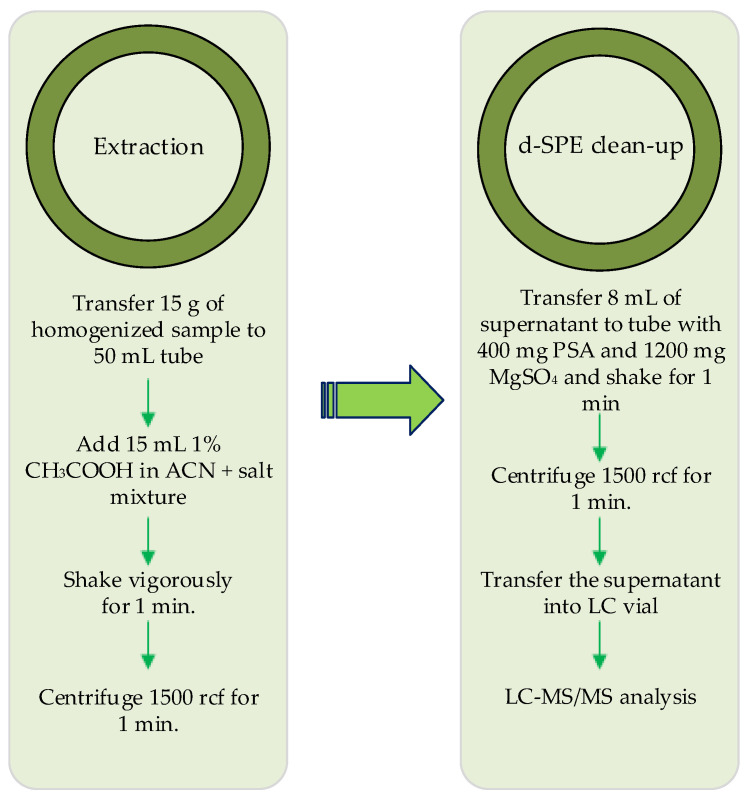
The procedure of the QuEChERS sample preparation method.

For sample preparation, the AOAC Official Method 2007.01 [39] QuEChERS procedure (Figure 1) was used. The procedure consists of two steps: extraction and d-SPE clean-up. In the first step, analytes were extracted from homogenized vine leaves (15 g) with ACN acidified with 1% CH_3_COOH and salts (6 g MgSO_4_ and 1.5 g CH_3_COONa). In the d-SPE clean-up process, a quantity of 1200 mg of MgSO_4_ was employed to eliminate excess water while 400 mg of PSA was used to remove potential interferences, such as carbohydrates, fatty acids, organic acids, lipids, and polar pigments, from the 8 mL extract.

### 2.4. LC-MS/MS Analysis

The identification and quantification of 512 pesticide residues in the samples of brined vine leaves were conducted using a LC-MS/MS system (Shimadzu LC-MS 8030 triple quadrupole mass spectrometer) coupled with a Shimadzu LC-20 series HPLC system (Shimadzu, Tokyo, Japan). Multi-residue compounds were separated using an SVEA Core C-18 reversed-phase column (3 × 100 mm, 2.6 µm particle size) (Nanologica AB, Södertälje, Sweden) at 40 °C with a gradient mode, as detailed in Table 1. The mobile phases comprised water–MeOH (98:2, *v*/*v*) (Mobile Phase A) and MeOH–water (98:2, *v*/*v*) (Mobile Phase B). Both phases included 0.1% CH_2_O_2_ and 5 mM HCOONH_4_. Ten microliters of injection volume were loaded onto the column.

Electrospray ionization (ESI) was employed with polarity switching. The mass spectrometer was operated in both positive and negative ionization modes with a spray voltage of 4.5 kV. The nebulizing gas flow rate was 3.04 L min^−1^ while the drying gas flow rate was 11 L min^−1^. The desolvation line temperature was maintained at 250 °C and the heat block temperature was set to 400 °C. Mass spectrometer data acquisition utilized the multiple reaction monitoring (MRM) mode, which recorded the transitions between the precursor ion and the two most abundant product ions for each target analyte, as outlined in the SANTE/11312/2021 Guidelines [40]. The LabSolutions software (version 5.96) was used for instrument control and data acquisition.

### 2.5. Method Validation

The method validation was conducted in-house according to SANTE/11312/2021 guidelines [40] for linearity, limits of quantification (LOQs), accuracy (recovery), precision (repeatability and within-laboratory reproducibility, expressed as relative standard deviation, RSD%), and measurement uncertainty. Matrix-matched standards were utilized to mitigate matrix effects and ensure accurate quantification. Linearity was assessed by examining matrix-matched calibration curves using spiked blank samples at concentrations of 0.005, 0.010, 0.025, 0.050, and 0.25 mg kg^−1^. The coefficients of determination (*R*^2^) for each compound were calculated. The accuracy, precision, and LOQs were determined through recovery trials. To assess acceptable accuracy (70–120%) and precision (RSD ≤ 20%), blank samples were spiked with mixed standard solutions at concentrations of 0.01 and 0.05 mg kg^−1^. The spiked materials were then evaluated in accordance with the method protocol and the target analytes were quantified using the matrix-matched calibration curves. LOQs were calculated using the standard deviation (SD) of a blank sample matrix fortified with pesticide mix at the lowest spiking level of 0.01 mg kg^−1^. Repeatability and within-laboratory reproducibility were assessed by conducting at least five repetitions to calculate intra-day (*n* = 5) and inter-day (*n* = 15, over three consecutive days) RSDs%. The reproducibility and trueness parameters were considered to calculate expanded measurement uncertainty (⋃′), as described in the EURACHEM guidelines [41].

## 3. Results and Discussion

### 3.1. Method Validation Data

The target residues were identified by comparing retention time and ion ratio data to reference standards and confirmed to be within the European Commission’s tolerance limit. The method’s linearity was tested using matrix-matched calibration curves. With calibration ranges from 0.005 to 0.25 mg kg^−1^, all residues exhibited a good linear response with *R*^2^ > 0.99 and all residuals fell within the 20% tolerance of the SANTE criteria. Comparing the LOQ values determined in the vine leaf matrix (0.01 mg kg^−1^ or lower) to the legally permitted levels indicates that they are applicable to all target analytes for monitoring the residue levels in vine leaves. The recovery, precision, and measurement uncertainty for all 512 pesticides assessed in this study are shown in Table S1. In order to determine recovery and precision with regard to repeatability and reproducibility conditions, blank samples of vine leaves fortified at two different mixed residue standards (0.01 and 0.05 mg kg^−1^) were subject to analysis. The average recovery values were between 75% and 117% at 0.01 mg kg^−1^ and between 83% and 113% at 0.05 mg kg^−1^. All recovery values met the acceptability requirements (recovery 70–120%) outlined in the SANTE Guideline. Within fortification levels, relative repeatability standard deviations between 0.7% and 19.4% and relative reproducibility standard deviations between 0.7% and 19.9 were obtained, which are an indication of a good precision of the method. The extended measurement uncertainty (6.6–35%), which included trueness and reproducibility, was less than 50% for all residues, as required by the SANTE Guidelines.

### 3.2. Pesticide Residues in Vine Leaves

During May 2022 and June 2023, a total of 766 brined vine leaf samples from Turkey were tested for the presence of 512 pesticide residues. Conducting surveillance of pesticide residues over more than one year yields a more reliable and comprehensive collection of data as it considers potential fluctuations in pesticide usage and fruit quality across multiple harvesting periods. Climatic conditions, pest prevalence, and farmer behaviors are among the variables that can cause annual fluctuations in agricultural practices, including pesticide usage. By conducting a consecutive two-year sampling of vine leaves, the research could more effectively ascertain the general pattern and regularity of pesticide contamination within the area.

In 23.5% of the samples analyzed, at least one pesticide residue was identified while no pesticide residue was detected in the remaining 586 samples. A total of forty-three distinct pesticides were identified in the brined vine leaf samples supplied by eighty-two different producers, comprising twenty-nine fungicides, eleven insecticides, two herbicides, and one acaricide. Of the forty-three active chemicals identified in vine leaves, eight were not authorized in the EU: chloridazon, indoxacarb, flutriafol, propiconazole, thiacloprid, thiamethoxam, thiophanate-methyl, and triadimenol.

Three-hundred and thirty-seven samples of brined vine leaves were examined in 2022. The distribution and concentration of residues detected in these samples are summarized in Table 2. Two-hundred and ninety-one samples, accounting for 86.4% of the total, did not contain any measurable residues. Out of the brined vine leaf samples analyzed, 13.6% were found to have at least one pesticide residue. However, 32 of these samples (9.5%) had pesticide levels that were above the MRLs.

**Figure 2:**
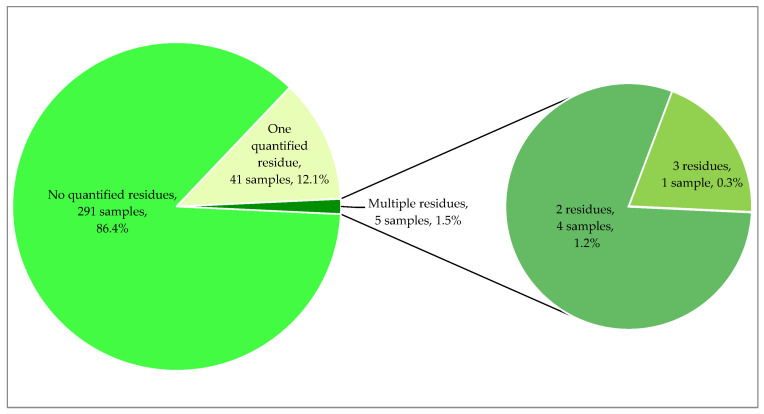
Percentage of vine leaf samples from 2022 without any residues or with residues.

In 493 (96.3% of the target analytes) out of the assessed 512 active substances, quantifiable levels were not detected in any sample collected in 2022. On the other hand, for 16 substances (3.1%), residues above the valid MRLs were detected. Although approved pesticides accounted for the majority of recorded residues (fifteen pesticides), four non-approved pesticides were individually detected: indoxacarb, thiamethoxam, thiophanate-methyl, and triadimenol. Only 1.5% of all vine leaf samples (five samples) contained more than one substance in quantifiable concentrations; whereas, forty-one samples contained one residue. The percentage distribution of the number of quantified residues in individual brined vine leaf samples is presented in Figure 2.

In 2022, cypermethrin was the most frequently identified residue in vine leaves, with a detection rate of 5.6%. Cypermethrin is a synthetic pyrethroid insecticide chemically synthesized from pyrethrin, a naturally occurring component obtained from the *Chrysanthemum* spp. flower. It is extensively utilized in agricultural and veterinary insecticides to manage a variety of insects, including cockroaches, mosquitoes, lice, ticks, and mites [42]. Cypermethrin is widely used as an insecticide against the European grapevine moth, *Lobesia botrana* (Lepidoptera: Tortricidae), in vineyards in Turkey [43]. Following a six-year approval extension, the EU awarded re-approval in February 2022 for a seven-year period, until 2029 [44]. The concentration of cypermethrin in vine leaves from 2022 ranged from 0.011 to 0.458 mg kg^−1^, with a mean value of 0.108 mg kg^−1^. In 10 samples, the residue concentrations of cypermethrin exceeded the MRL of 0.05 mg kg^−1^.

Residues of metalaxyl (1.8%) and deltamethrin (1.5%) were detected in over 1% of the vine leaves from 2022, with concentration ranges of 0.011–0.222 mg kg^−1^ and 0.010–0.027 mg kg^−1^, respectively. While the MRL was exceeded for metalaxyl in all detected samples, no MRL exceedance was identified for deltamethrin. Less frequently found residues were lambda-cyhalothrin (0.9%), metrafenone (0.9%), boscalid (0.6%), difenoconazole (0.6%), acetamiprid (0.3%), azoxystrobin (0.3%), indoxacarb (0.3%), kresoxim-methyl (0.3%), penconazole (0.3%), propamocarb (0.3%), pyraclostrobin (0.3%), pyrimethanil (0.3%), tebuconazole (0.3%), thiamethoxam (0.3%), thiophanate-methyl (0.3%), and triadimenol (0.3%).

In 2023, a total of 429 brined vine leaf samples were analyzed. The distribution and concentration of residues detected in these samples are shown in Table 3. The quantification rate of pesticides in vine leaf samples from 2023 (31.2%) exhibited a more than two-fold increase compared to the 2022 results (13.6%). There were no detectable residues in 68.8% of the vine leaf samples. Compared to the previous year, the MRL exceedance rate for vine leaves rose significantly. In 2023, 108 samples (25.2%) had MRL exceedances; whereas, 26 samples (6.0% of the total) had detectable pesticides within legally allowed quantities. The residues that exceeded the MRLs were associated with 29 specific substances, with lambda-cyhalothrin being the most frequently detected.

**Figure 3:**
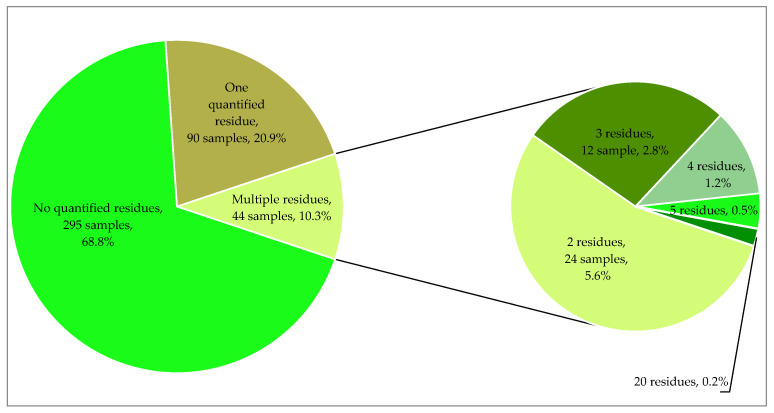
Percentage of vine leaf samples from 2023 without any residues or with residues.

The number of residues detected in vine leaves from the 2023 harvest season was more than two-fold higher than that of samples from the previous year. Specifically, in 2023, forty different substances were identified in quantifiable concentrations, six of which were non-approved in the EU: chloridazon, flutriafol, indoxacarb, propiconazole, thiacloprid, and thiophanate-methyl. While most detected samples contained only one residue, with an incidence rate of 20.9%, 10.3% of the samples (44 samples) had multiple residues. Remarkably, individual vine leaf samples from 2023 showed the presence of up to 20 residues (Figure 3). Out of the forty-four samples that had multiple residues, 5.6% (twenty-four samples) had two residues, 2.8% (twelve samples) had three residues, 1.2% (five samples) had four residues, 0.5% (two samples) had five residues, and 0.2% (one sample) had twenty residues. This represents a significant increase in the rate of multiple residues for vine leaves in 2023 (10.3%) compared to 2022 (1.5%).

Similar to the previous year, pyrethroids remained the most frequently quantified pesticide type in vine leaves from 2023. Specifically, the insecticides lambda-cyhalothrin and cypermethrin were among the most frequently detected compounds, with frequencies of 13.5% and 8.4%, respectively. The concentrations of lambda-cyhalothrin and cypermethrin in the samples analyzed in 2023 varied from 0.010 to 0.158 mg kg^−1^ and from 0.011 to 0.533 mg kg^−1^, with average concentrations of 0.040 and 0.079 mg kg^−1^, respectively. Of the 58 samples containing lambda-cyhalothrin, 55 of them exceeded the MRL of 0.01 mg kg^−1^. Regarding cypermethrin, 16 samples (3.7%) exceeded the MRL of 0.05 mg kg^−1^.

It should also be highlighted that higher quantification rates for both lambda-cyhalothrin (+12.6%) and cypermethrin (+2.8%) were recorded in vine leaves from 2023 compared with 2022. Among the more than 50 types of pyrethroids available on the pesticide market, lambda-cyhalothrin stands out as a common broad-spectrum non-systemic insecticide. Alongside cypermethrin, lambda-cyhalothrin has been widely employed for controlling *Lobesia botrana* [45], which is the primary pest that affects grape berries in vineyards across Turkey and Southern Europe. In a study conducted in Turkey, the half-life of lambda-cyhalothrin was determined to be 2.3 days in vine leaves [46]. This active substance was approved until 31 August 2026 [47].

In 2023, the quantification rate also increased for the fungicides metalaxyl (+4.5%) and pyraclostrobin (+3.2%), as well as for another pyrethroid insecticide, deltamethrin (+1.8%). The residues of metalaxyl, pyraclostrobin, and deltamethrin were detected in 6.3%, 3.5%, and 3.3% of the brined vine leaves, with concentrations ranging from 0.011 to 0.166 mg kg^−1^ (mean = 0.044 mg kg^−1^), 0.010 to 0.434 mg kg^−1^ (mean = 0.079 mg kg^−1^), and 0.010 to 0.078 mg kg^−1^ (0.023 mg kg^−1^), respectively. The MRL was exceeded for metalaxyl (in all detected samples) and pyraclostrobin (in 11 out of 15 samples); whereas, MRL exceedances were not observed for deltamethrin in brined vine leaves.

The pesticides ametocradin (1.9%), boscalid (1.6%), dimethomorph (1.4%), and pyrimethanil (1.4%) were quantified in more than 1% of the samples while the other 31 residues were detected sporadically. Three substances (propamocarb, thiamethoxam, and triadimenol), which were individually identified in 2022, were not detected in 2023.

The database of the Rapid Alert System for Food and Feed (RASFF) [48] corroborated the findings of the current investigation. Regarding vine leaves, Turkey and Egypt emerged as the most frequently notified countries of origin. In 2022, there were nineteen notifications of pesticide residues for vine leaves; among these, eleven (57.9%) originated from Turkey, six from Egypt (31.6%), one from Lebanon (5.3%), and one from the United Arab Emirates (5.3%). Dithiocarbamates (seven notifications), lambda-cyhalothrin (six), carbendazim (five), metalaxyl (five), and chlorpyrifos/chlorpyrifos-methyl (five) were the most frequently reported residues in vine leaves in 2022. The number of notifications related to pesticide residues in vine leaves increased to 20 in 2023. Conversely, in 2023, Egypt was the most notified country of origin (12 notifications, 60%). There was a 22.9% decrease in notifications of pesticide residues in vine leaves from Turkey in 2023 (seven notifications) compared to 2022. In 2023, the most notified residues in vine leaves were carbendazim (ten notifications), chlorpyrifos/chlorpyrifos-methyl (nine), acetamiprid (eight), bifenthrin (seven), lambda-cyhalothrin (seven), azoxystrobin (six), boscalid (six), metalaxyl (five), difenoconazole (five), and propiconazole (five).

The frequency rate of residues found in the present study is much lower than that reported earlier by Özata [25], who observed 14 out of 16 fresh grape leaves (87.5%) and 29 out of 32 brined vine leaves (90.6%) collected from Tokat province, Turkey that contained one or multiple residues. The most frequently identified pesticides in fresh vine leaves were trichlorfon (71.4%), dithianon (50%), and cypermethrin (28.6%); whereas, triadimenol (46.9%), metalaxyl (34.4%), and azoxystrobin (31.3%) were the three most commonly found residues in vine leaves in brine. In another Turkish study, a total of 232 samples of vine leaves were collected from retail stores in 2017 and analyzed for 318 pesticides. In 36.6% of the samples, 42 different pesticides were detected in measurable concentrations; whereas, 52 samples (22.4%) contained residues above the MRLs [26]. More recently, 15 pickled vine leaf samples collected from local markets in Tokat province, Turkey, were monitored for the presence of 243 pesticides. All samples contained at least one residue. Among the thirteen recorded pesticides, ethiofencarb (100%, 0.008–0.011 mg kg^−1^), isocarbofos (40%, 0.015–0.054 mg kg^−1^), and cyhalothrin (33.3%, 0.020–0.316 mg kg^−1^) were the three most common residues in pickled vine leaves [28]. This incidence rate is much higher than that obtained in the present study. The variation may be attributed to factors like climatic conditions, pest abundance, and farmer practices, which fluctuate annually and might affect the utilization of pesticides.

Pesticide residues in vine leaves represent one of the most significant food safety concerns, not only in Turkey but also in Egypt, prompting worldwide public health worries, particularly in Europe. In a survey conducted in Egypt from 2012 to 2013, forty-eight samples of vine leaves were monitored for the presence of twenty-six pesticides (twenty-one organophosphates and five carbamates). The residues of oxamyl (47.9%), chlorpyrifos (45.8%), diazinon (41.7%), chlorpyrifos-methyl (39.6%), methomyl (39.6%), and carbofuran (33.3%) were the most frequently detected [49]. In 2021 in Egypt, Hamzawy [50] analyzed seventy-eight samples of vine leaves collected from local markets and reported that only one was free of any pesticides (98.7% incidence). MRL exceedances were noted for 36 residues. Among the more than 400 pesticides, atrazine was recorded with the highest frequency (74%), followed by boscalid (68%), propiconazole (55%), lambda-cyhalothrin (44%), and myclobutanil (42%).

Vine leaves can be preserved using various methods, including brining, drying, bleaching, freezing, and pickling. The choice of preservation method depends on personal preference, culinary application, and the desired characteristics of the preserved vine leaves. In a study in Lebanon, twenty-four samples of vine leaves preserved with three different methods, collected from the local markets, were monitored for thirty-three pesticide residues. The dried preserved vine leaves exhibited the highest levels of residues compared to vine leaves in brine and stuffed vine leaves. Moreover, systemic pesticide residues were more commonly quantified compared to contact pesticides. Chlorpyrifos, fenazaquin, lambda-cyhalothrin, and carbendazim were reported as the most frequently detected residues in vine leaves, predominantly in the samples of dried preserved leaves [2]. In another study, the effects of preservation methods on the pesticide residue levels in vine leaves were assessed. The residue levels in dried preserved vine leaves were found to be significantly higher than those preserved in brine at two different temperatures (26.5 and 80 °C). The concentration of pesticide residues in vine leaves preserved in brine exhibited a reduction of 69–73% at 26.5 °C and 73–91% at 80 °C compared to the samples of dried preserved leaves [27].

The presence of pesticide residues in food, including vine leaves, raises concerns regarding potential health implications for consumers. Each of the pesticides detected in vine leaves carries distinct toxicological profiles and potential health risks. Studies have linked exposure to synthetic pyrethroids with adverse health effects, including neurotoxicity, respiratory irritation, and allergic reactions. Additionally, chronic exposure to these compounds has been associated with developmental and reproductive toxicity in animal studies [51]. Lambda-cyhalothrin and cypermethrin belong to the class of synthetic pyrethroids, commonly used as insecticides in vine leaves. The present data are too insufficient to define the potential endocrine activities of lambda-cyhalothrin; although, the most sensitive systemic effect observed was neurotoxicity, resulting in decreased motor activity [52]. Cypermethrin possesses endocrine-mediated activity as well; however, the possibility of endocrine disruption has not been definitively determined [53]. Deltamethrin, another synthetic pyrethroid insecticide, shares similar toxicity profiles with lambda-cyhalothrin and cypermethrin. Exposure to deltamethrin has been associated with neurotoxic effects, including tremors, convulsions, and sensory disturbances [54]. Another more frequently detected residue, metalaxyl, has low to moderate acute toxicity by oral, dermal, or inhalation exposure; whereas, it does not possess genotoxic, carcinogenic, or neurotoxic activity [55]. It is essential to recognize that the health risks associated with pesticide residues in food are influenced by various factors, including pesticide toxicity, exposure levels, and individual susceptibility. Moreover, cumulative exposure to multiple pesticides, as observed in our study, may potentiate adverse health effects through synergistic or additive mechanisms [56].

## 4. Conclusions

This study aimed to determine multi-class pesticide residue levels in brined vine leaves from Turkey using the QuEChERS method followed by LC-MS/MS analysis. The analytical method demonstrated fit-for-purpose performance in terms of linearity, accuracy, precision, and measurement uncertainty. Pesticide residues were detected in 23.5% of the samples analyzed while no pesticide residue was observed in the remaining 586 samples. There were forty-three different pesticides identified in the samples of brined vine leaves, comprising twenty-nine fungicides, eleven insecticides, two herbicides, and one acaricide. Compared to the 2022 results, both the quantification rates of pesticides and the MRL exceedance rates in brined vine leaves significantly increased in 2023. The number of detected residues in 2023 (*n* = 40) more than doubled compared to 2022 (*n* = 19). Among the pesticides analyzed, the most frequently detected were lambda-cyhalothrin (8.0%), cypermethrin (7.2%), metalaxyl (4.3%), deltamethrin (2.5%), and pyraclostrobin (2.1%). The prevalence of lambda-cyhalothrin in the RASFF warnings issued by European countries is consistent with its high occurrence in vine leaves from Turkey. Out of forty-three compounds detected in the vine leaf samples, eight were unauthorized in the EU. These findings provided valuable information into the current pesticide contamination levels and quality assessment of vine leaves from Turkey. The detection of unauthorized compounds in vine leaf samples raises significant regulatory concerns as it underscores potential gaps in regulatory oversight and enforcement mechanisms. Such findings highlight the need for stricter monitoring and enforcement measures to ensure compliance with established regulatory frameworks. Moreover, the presence of unauthorized compounds may pose risks to consumer health. As a result, there is an urgent need for comprehensive risk evaluations and mitigation techniques to protect consumer health and wellbeing. Furthermore, the detection of unauthorized compounds may have implications for international trade as it may lead to trade disruptions and barriers due to non-compliance with regulatory standards. The Ministry of Agriculture and Forestry of Turkey should consider strengthening its efforts by organizing multiple extension training sessions for vineyard producers, with a focus on vine leaf production.

## Figures and Tables

**Table 1:** Gradient conditions for LC.

Time (min)	Mobile Phase A% ^a^	Mobile Phase B% ^b^	Flow (mL min^−1^)
0.0	80	20	0.6
0.2	80	20	0.6
1.5	30	70	0.6
5.0	5	95	0.6
9.0	5	95	0.6
9.1	80	20	0.6
10.0	80	20	0.6

^a^ Water–MeOH (98:2, *v*/*v*) with 0.1% CH_2_O_2_ and 5 mM HCOONH_4_. ^b^ MeOH–water (98:2, *v*/*v*) with 0.1% CH_2_O_2_ and 5 mM HCOONH_4_.

**Table 2:** The distribution and concentration of pesticides in brined vine leaves in 2022.

Pesticide	Pesticide Type	EU MRL (mg kg^−1^)	% of Samples <LOQ	% of Samples LOQ-MRL	% of Samples >MRL	Range (mg kg^−1^)	
						Min.–Max.	Mean
Acetamiprid	IN ^a^	0.01	99.7	-	0.3	0.083	0.083
Azoxystrobin	FU ^b^	0.01	99.7	-	0.3	0.735	0.735
Boscalid	FU	0.01	99.4	-	0.6	0.147–0.286	0.217
Cypermethrin	IN	0.05	94.4	2.7	3.0	0.011–0.458	0.108
Deltamethrin	IN	2.0	98.5	1.5	-	0.010–0.027	0.016
Difenoconazole	FU	0.05	99.4	0.3	0.3	0.044–0.603	0.324
Indoxacarb *	IN	0.02	99.7	-	0.3	0.089	0.089
Kresoxim-methyl	FU	15.0	99.7	0.3	-	0.010	0.010
Lambda-cyhalothrin	IN	0.01	99.1	-	0.9	0.040–0.248	0.113
Metalaxyl	FU	0.01	98.2	-	1.8	0.011–0.222	0.056
Metrafenone	FU	0.01	99.1	-	0.9	0.012–0.259	0.123
Penconazole	FU	0.01	99.7	-	0.3	0.011	0.011
Propamocarb	FU	0.01	99.7	-	0.3	0.098	0.098
Pyrimethanil	FU	0.01	99.7	-	0.3	0.011	0.011
Pyraclostrobin	FU	0.02	99.7	-	0.3	0.062	0.062
Tebuconazole	FU	0.02	99.7	-	0.3	0.040	0.040
Thiamethoxam *	IN	0.01	99.7	-	0.3	0.087	0.087
Thiophanate-methyl *	FU	0.10	99.7	0.3	-	0.027	0.027
Triadimenol *	FU	0.01	99.7	-	0.3	0.014	0.014

^a^ IN: insecticide. ^b^ FU: fungicide. * Not approved in the EU.

**Table 3:** The distribution and concentration of pesticides in brined vine leaves in 2023.

Pesticide	Pesticide Type	EU MRL (mg kg^−1^)	% of Samples <LOQ	% of Samples LOQ-MRL	% of Samples >MRL	Range (mg kg^−1^)	
						Min.–Max.	Mean
Acetamiprid	IN ^a^	0.01	99.5	-	0.5	0.011–0.088	0.050
Ametoctradin	FU	50.0	98.1	1.9	-	0.010–6.319	1.477
Azoxystrobin	FU ^b^	0.01	99.5	-	0.5	0.032–0.070	0.051
Boscalid	FU	0.01	98.4	-	1.6	0.011–0.232	0.071
Chloridazon *	HB ^c^	0.03	99.5	0.2	0.2	0.017–0.036	0.027
Cymoxanil	FU	0.01	99.8	-	0.2	4.121	4.121
Cypermethrin	IN	0.05	91.6	4.7	3.7	0.011–0.533	0.079
Cyprodinil	FU	0.02	99.1	0.2	0.7	0.014–0.314	0.104
Deltamethrin	IN	2.0	96.7	3.3	-	0.010–0.078	0.023
Difenoconazole	FU	0.05	99.8	0.2	-	0.018	0.018
Dimethomorph	FU	0.01	98.6	-	1.4	0.020–0.554	0.113
Fluazifop-P	HB	0.01	99.5	-	0.5	0.012–0.195	0.104
Fludioxonil	FU	0.01	99.8	-	0.2	0.063	0.063
Fluopicolide	FU	30.0	99.8	0.2	-	0.948	0.948
Fluopyram	FU/NE ^d^	0.01	99.5	-	0.5	0.018–0.032	0.025
Flutriafol *	FU	0.01	99.5	-	0.5	0.035–0.104	0.070
Fluxapyroxad	FU	0.01	99.8	-	0.2	0.111	0.111
Hexythiazox	AC ^e^	0.01	99.5	-	0.5	0.013–0.017	0.015
Indoxacarb *	IN	0.02	99.8	-	0.2	0.026	0.026
Iprovalicarb	FU	0.01	99.8	-	0.2	0.468	0.468
Kresoxim-methyl	FU	15.0	99.5	0.5	-	0.033–0.152	0.093
Lambda-cyhalothrin	IN	0.01	86.5	0.7	12.8	0.010–0.158	0.040
Malathion	IN/AC	0.02	99.1	0.5	0.5	0.011–0.148	0.076
Mandipropamid	FU	25.0	99.5	0.5	-	0.074–1.838	0.956
Metalaxyl	FU	0.01	93.7	-	6.3	0.011–0.166	0.044
Metrafenone	FU	0.01	99.8	-	0.2	0.027	0.027
Penconazole	FU	0.01	99.8	-	0.2	0.014	0.014
Propiconazole *	FU	0.01	99.8	-	0.2	0.020	0.020
Proquinazid	FU	0.01	99.8	-	0.2	0.013	0.013
Pyrimethanil	FU	0.01	98.6	0.5	0.9	0.010–0.065	0.024
Pyraclostrobin	FU	0.02	96.5	0.9	2.6	0.010–0.434	0.079
Spinetoram	IN	0.02	99.8	0.2	-	0.011	0.011
Spirotetramat	IN	0.02	99.8	0.2	-	0.016	0.016
Spiroxamine	FU	0.01	99.8	-	0.2	0.591	0.591
Tau-fluvalinate	IN	0.01	99.8	-	0.2	0.013	0.013
Tebuconazole	FU	0.02	99.8	0.2	-	0.010	0.010
Thiacloprid *	IN	0.01	99.8	-	0.2	0.013	0.013
Thiophanate-methyl	FU	0.10	99.8	0.2	-	0.025	0.025
Trifloxistrobin	FU	0.01	99.8	0.2	-	0.010	0.010
Zoxamide	FU	0.02	99.5	-	0.5	0.133–1.026	0.580

^a^ IN: insecticide. ^b^ FU: fungicide. ^c^ HB: herbicide. ^d^ NE: nematicide. ^e^ AC: acaricide. * Not approved in the EU.

## Data Availability

The original contributions presented in the study are included in the article/Supplementary Materials, further inquiries can be directed to the corresponding author.

## Supplementary Materials

The following supporting information can be downloaded at: https://www.mdpi.com/article/10.3390/foods13060909/s1, Table S1: The in-house validation data for 512 residues in brined vine leaves by LC-MS/MS.
