# Supplementary material for: Determination of Pesticide Residues in Vine Leaves Using the QuEChERS Method and Liquid Chromatography-Tandem Mass Spectrometry
Source: Foods. 2024 Mar 17;13(6):909. doi: 10.3390/foods13060909 (PMC10969690; doi:10.3390/foods13060909)
Supplement: Supplementary file 1 [file foods-13-00909-s001.zip › foods-2919000-supplementary.pdf]

Supplementary material

Table S1. The in-house validation data for 512 residues in brined vine leaves by LC-MS/MS.

Pesticide	LOQ (mg kg^{-1})	Recovery (%)		Repeatability (%RSD$_r$, n=5)		Within-laboratory reproducibility (%RSD$_R$, n=15)		U (%)
		0.01 mg kg^{-1}	0.05 mg kg^{-1}	0.01 mg kg^{-1}	0.05 mg kg^{-1}	0.01 mg kg^{-1}	0.05 mg kg^{-1}	
2,4,5-T	0.003	112	106	4.8	1.8	6.9	8.2	16.6
2,4,5-TP (Fenoprop)	0.002	91	93	5.5	4.7	10.9	3.8	19.1
2,4,6 trichlorophenol	0.007	97	92	7.4	3.2	18.6	2.9	26.8
2,4-D	0.004	110	99	2.7	3.1	13.0	4.8	20.2
2,4-DB	0.002	106	103	5.9	3.8	12.2	7.1	20.6
2,4-Dimethylaniline	0.006	90	96	7.9	3.3	15.3	3.0	23.1
2,4-DP (Dichlorprop)	0.003	108	104	2.6	3.2	5.4	6.3	12.7
2-Naphthyloxyacetic acid	0.004	111	106	5.4	1.6	3.2	3.9	9.9
4-Chlorobenzoic acid	0.005	104	101	4.4	7.2	14.0	6.4	22.6
4-CPA	0.005	91	103	6.6	3.3	9.6	9.5	20.1
Abamectin	0.006	83	103	10.0	2.4	12.0	8.9	22.2
Acephate	0.003	107	107	6.7	2.1	14.8	2.7	21.8
Acetamiprid	0.003	92	105	8.3	4.0	10.9	6.9	19.1
Acetochlor	0.007	101	106	7.1	2.3	13.2	1.9	19.8
Acibenzolar-S-methyl	0.005	86	111	14.2	5.0	8.7	3.5	15.2
Aclonifen	0.004	89	106	11.5	3.8	2.6	4.4	10.4
Alachlor	0.003	110	104	6.7	3.1	8.4	5.8	15.3
Aldicarb	0.005	115	101	2.2	5.5	19.3	3.3	28.0
Aldicarb sulfone	0.006	111	103	3.0	6.0	4.1	7.6	14.0
Aldicarb sulfoxide	0.002	98	99	8.1	3.9	8.1	5.1	14.4
Ametoctradin	0.006	108	92	2.9	1.3	14.3	6.7	22.8
Amidosulfuron	0.008	93	95	13.0	5.3	17.7	4.4	27.1
Aminocarb	0.006	84	103	15.2	2.9	17.1	5.3	26.1
Aminopyralid	0.003	100	106	8.3	2.3	5.6	9.5	16.3
Amisulbrom	0.008	84	107	11.5	4.7	14.7	5.6	23.5
Amitraz	0.002	112	102	4.9	5.9	15.9	11.5	28.0
Anilazine	0.004	92	93	3.5	1.3	9.1	4.2	14.8
Anilofos	0.003	107	102	9.2	6.0	11.7	8.6	21.1
Aramite	0.007	98	97	13.2	2.8	8.8	6.3	16.8

Atrazine	0.004	98	102	10.0	3.1	7.7	14.3	23.8
Atrazine-desethyl-desisopropyl	0.010	107	100	12.4	2.5	8.0	8.3	17.2
Atrazine-desethyl	0.003	106	107	6.7	2.9	15.2	4.9	23.5
Azaconazole	0.006	110	105	18.7	6.7	17.4	6.0	27.4
Azadirachtin A	0.009	104	93	12.1	2.7	18.0	6.1	27.9
Azamethiphos	0.007	105	96	9.7	0.8	6.0	5.4	12.5
Azimsulfuron	0.006	94	99	16.5	3.3	8.1	4.2	15.1
Azinphos-ethyl	0.008	106	96	9.1	1.4	9.7	2.9	15.6
Azinphos-methyl	0.004	106	110	9.2	0.7	10.3	5.4	17.3
Azoxystrobin	0.005	99	104	11.2	5.7	14.0	10.3	25.4
Barban	0.006	100	105	5.3	6.4	13.5	8.1	24.9
Benalaxyl	0.004	114	101	2.3	3.9	18.2	9.4	29.3
Benazolin	0.005	99	104	16.1	2.2	16.1	3.7	25.1
Bendiocarb	0.007	105	104	13.3	5.4	9.4	8.4	19.3
Benflubutamid	0.004	80	96	12.7	5.3	17.7	5.4	28.1
Benfluralin	0.005	102	91	8.1	0.7	8.9	5.6	15.9
Benfuracarb	0.004	92	104	5.3	3.4	10.1	12.7	23.8
Benomyl	0.003	84	92	15.2	3.3	12.4	8.9	22.7
Benoxacor	0.008	97	109	4.1	3.4	15.9	5.7	24.6
Bensulfuron-methyl	0.006	101	101	6.8	3.1	13.0	7.4	21.9
Bentazone	0.005	97	96	13.9	5.8	5.8	4.6	12.3
Bicyclopyrone	0.003	93	104	4.9	2.8	5.5	5.6	12.1
Bifenazate	0.002	101	105	9.7	3.5	7.1	6.7	14.9
Bifenox	0.006	93	106	17.8	4.4	11.0	4.1	18.6
Bifenthrin	0.004	103	104	4.6	3.8	9.4	9.9	19.6
Bioresmethrin	0.007	104	102	9.5	1.6	4.0	5.6	10.7
Bispyribac sodium	0.003	94	99	7.9	2.2	8.8	3.0	13.7
Bitertanol	0.005	105	101	8.7	3.1	5.1	3.0	10.5
Bixafen	0.006	94	94	8.3	1.3	5.2	9.5	16.0
Boscalid	0.004	93	100	13.9	5.9	4.8	9.6	16.7
Bromacil	0.005	104	104	9.1	6.8	16.8	7.0	26.2
Bromophos-ethyl	0.007	104	97	6.2	4.0	7.7	5.3	14.3
Bromoxynil	0.004	106	102	7.7	5.7	9.2	4.6	16.0
Bromuconazole	0.005	86	102	9.8	8.1	11.3	8.8	21.3
Bupirimate	0.005	102	105	12.0	6.2	6.7	4.9	13.3
Buprofezine	0.005	93	103	18.1	4.7	4.6	13.3	21.2

Butafenacil	0.008	97	103	10.6	3.0	14.3	5.9	22.8
Butocarboxim	0.003	95	99	10.8	5.2	15.8	4.4	24.2
Butocarboxim-sulfoxide	0.004	76	95	3.6	2.3	5.3	10.4	16.7
Butralin	0.005	101	98	7.9	1.6	19.1	8.4	30.0
Buturon	0.006	110	107	7.6	3.6	18.5	14.8	33.9
Butylate	0.005	93	98	13.6	3.3	18.6	8.2	29.4
Cadusafos	0.002	109	104	3.7	4.1	15.3	9.6	25.9
Carbaryl	0.003	92	104	15.6	7.5	9.5	9.0	19.8
Carbendazim/Benomyl	0.002	96	96	4.4	2.7	6.5	8.2	15.2
Carbetamide	0.004	90	105	5.3	6.7	8.9	10.2	19.6
Carbofuran	0.002	106	106	9.9	2.5	18.5	6.6	28.3
Carbofuran-3-hydroxy	0.003	98	105	14.1	5.9	5.2	9.9	17.1
Carbosulfan	0.007	89	104	13.6	5.9	14.8	10.8	26.8
Carboxin	0.003	82	105	12.5	3.6	12.8	8.3	22.7
Carfentrazone-ethyl	0.007	85	97	12.3	9.6	12.6	4.3	20.3
Chlorantraniliprole	0.007	98	102	16.3	5.5	12.0	7.2	21.7
Chlorbromuron	0.005	106	105	13.6	7.4	17.5	8.7	28.9
Chlorbufam	0.007	93	101	13.4	7.6	7.7	4.0	14.9
Chlorfenvinphos	0.002	109	104	5.4	3.9	12.7	4.6	20.2
Chlorfluazuron	0.004	97	101	7.3	4.7	8.3	3.8	14.3
Chloridazon	0.004	93	106	7.6	2.3	4.4	7.9	13.8
Chlormequat chloride	0.005	92	97	14.4	2.6	17.3	8.1	27.8
Chlorotoluron	0.006	94	92	12.5	2.3	18.7	3.7	27.6
Chloroxuron	0.006	115	100	4.3	2.9	3.5	9.9	15.3
Chlorpropham	0.003	100	105	5.4	4.8	9.1	9.1	18.7
Chlorpyrifos	0.002	98	106	6.7	0.7	10.9	3.2	16.6
Chlorpyrifos methyl	0.002	93	97	7.4	2.0	8.3	5.0	14.3
Chlorsulfuron	0.005	115	100	2.2	5.8	17.5	10.1	29.2
Chlortal-dimethyl	0.006	98	94	13.2	3.3	16.6	9.1	27.6
Cinidon-ethyl	0.006	115	106	5.0	2.4	12.0	5.8	19.9
Clethodim	0.004	100	93	11.7	2.4	13.7	10.3	24.9
Climbazole	0.007	110	109	8.0	3.8	9.2	7.2	17.6
Clodinafop-propargyl ester	0.004	78	107	8.5	1.3	7.3	3.0	12.0
Clofentezine	0.004	94	100	6.7	4.5	4.7	7.0	13.0
Clomazone	0.007	114	105	7.5	4.3	18.7	10.2	30.8
Clopyralid	0.007	93	95	6.4	4.4	15.5	6.7	24.6

Cloquintocet-methylhexyl ester	0.006	105	92	8.8	1.4	8.8	4.2	15.4
Clothianidin	0.002	108	102	7.7	1.8	17.9	3.2	26.4
Coumaphos	0.007	105	107	14.5	1.1	18.8	13.2	33.6
Cyanazine	0.007	102	108	11.0	4.0	12.1	6.1	20.5
Cyanophos	0.006	109	110	18.6	5.6	12.8	9.5	24.5
Cyantraniliprole	0.007	98	91	9.3	2.1	15.2	5.3	23.8
Cyazofamid	0.006	102	100	8.1	7.3	12.9	4.7	20.7
Cyclanilide	0.004	88	103	6.2	1.7	11.0	2.9	16.8
Cycloate	0.003	111	100	2.8	4.8	11.1	2.6	16.6
Cycloxydim	0.005	111	93	6.1	3.4	6.5	5.8	13.2
Cyflumetofen	0.001	98	92	9.3	2.8	9.1	7.1	17.2
Cyhalafop-buthyl	0.006	76	101	11.4	2.1	18.5	3.9	27.5
Cymoxanil	0.006	80	94	9.4	3.4	5.2	5.5	11.6
Cypermethrin (alpha+beta+teta+zeta)	0.004	96	104	8.6	3.1	9.1	7.5	18.0
Cyproconazole	0.006	102	102	14.2	2.5	11.4	8.8	21.8
Cyprodinil	0.004	107	104	4.9	4.8	8.8	8.5	18.2
Cyromazine	0.002	88	100	11.8	4.0	14.0	8.9	25.1
Daminozide	0.003	93	102	13.6	8.6	11.8	4.8	21.0
Dazomet	0.007	87	93	18.9	2.7	19.2	5.2	29.2
Deltamethrin	0.005	105	105	4.7	3.1	7.4	11.3	20.0
Demeton-S-methyl	0.003	109	101	7.2	6.0	6.0	7.3	14.8
Demeton-S-methyl sulfone	0.003	96	101	11.2	4.2	10.8	5.5	18.4
Demeton-S-methyl sulfoxide	0.002	91	104	16.3	4.1	10.9	9.8	21.8
Desmedipham	0.004	77	94	9.9	3.0	13.9	5.3	21.7
Desmetryn	0.009	111	93	16.9	2.6	14.6	3.4	22.9
Diafenthiuron	0.003	102	102	8.4	3.6	14.3	9.5	24.7
Dialifos	0.008	108	93	11.3	1.0	18.5	14.8	34.0
Diallate	0.008	94	105	11.6	2.9	9.5	4.6	15.8
Diazinon	0.004	114	104	4.2	4.4	18.4	4.7	27.8
Dicamba	0.004	115	98	2.4	4.0	15.5	2.9	23.6
Dichlofenthion	0.004	104	98	13.0	5.3	9.0	3.1	15.2
Dichlofluanid	0.001	109	106	6.2	3.8	9.7	7.0	18.1
Dichlorvos (DDVP)	0.005	92	103	12.0	2.9	14.7	6.2	23.4
Diclobutrazol	0.007	93	99	8.1	4.3	10.5	3.1	16.5
Diclofop-methyl	0.008	110	105	5.0	1.9	13.7	10.0	24.5

Dicrotophos	0.006	97	97	7.4	1.2	16.7	5.1	25.0
Diethofencarb	0.002	79	96	5.5	5.5	17.3	3.0	25.6
Difenoconazole	0.002	109	104	4.9	5.6	14.4	13.0	27.9
Diflubenzuron	0.006	101	101	12.9	6.6	13.4	16.1	30.4
Diflufenican	0.003	102	101	4.9	6.0	9.0	6.4	16.5
Dimethachlor	0.005	98	102	17.2	7.4	4.3	2.7	10.7
Dimethenamid	0.004	102	103	7.2	5.0	4.5	4.7	10.3
Dimethoate	0.003	92	106	8.5	3.4	4.6	7.4	13.1
Dimethomorph	0.007	87	102	15.3	4.6	6.4	6.8	14.7
Dimoxystrobin	0.005	93	98	18.6	4.4	16.4	9.7	28.4
Diniconazole	0.003	90	103	5.8	4.3	13.6	6.9	21.9
Dinocap	0.007	85	103	6.5	5.4	16.8	3.3	24.7
Dinotefuran	0.004	88	100	8.6	6.8	16.4	4.8	24.6
Dinoterb	0.006	85	90	3.6	1.7	18.2	14.3	33.0
Dioxacarb	0.002	93	96	6.2	2.9	9.9	9.7	19.9
Dioxathion	0.009	111	98	9.8	9.1	10.3	4.2	17.0
Diphenamid	0.006	91	100	14.9	1.1	10.9	11.6	23.7
Diphenylamine	0.006	99	104	10.1	5.4	5.5	10.2	17.5
Disulfoton	0.003	80	101	9.0	5.6	0.7	1.9	6.6
Disulfoton sulfone	0.004	95	97	8.7	9.3	5.0	4.2	11.5
Ditalimfos	0.007	103	109	15.1	2.2	17.2	4.2	25.8
Dithianon	0.004	106	104	10.5	3.5	16.8	6.3	26.2
Diuron	0.005	92	96	11.0	2.2	8.4	3.4	14.8
DMST	0.004	106	102	8.7	7.8	8.1	4.2	15.0
DNOC	0.006	90	101	10.2	6.0	9.5	5.6	16.8
Dodine	0.005	108	88	9.4	3.7	16.1	9.2	26.6
Emamectin benzoate	0.006	102	89	5.4	0.8	19.2	6.8	29.4
Endosulfan sulfate	0.004	103	101	10.6	5.3	15.6	5.8	24.5
Epoxiconazole	0.003	106	99	6.7	4.3	15.3	11.3	27.4
EPTC	0.008	83	97	11.0	3.6	15.2	4.1	22.6
Esfenvalerate	0.005	95	89	4.2	2.6	13.3	3.6	19.8
Etaconazole	0.005	94	92	15.2	2.8	10.6	2.3	17.6
Ethametsulfuron methyl	0.003	89	93	7.3	1.8	6.3	3.8	11.1
Ethiofencarb	0.004	109	97	3.5	4.0	19.6	12.9	33.4
Ethiofencarb-sulfone	0.007	105	102	10.3	2.4	8.4	6.0	16.4
Ethiofencarb-sulfoxide	0.009	107	97	11.2	3.2	11.8	4.6	18.9

Ethion	0.003	108	102	3.5	4.7	3.9	8.3	14.3
Ethiprole	0.005	75	106	10.6	5.7	19.3	9.3	31.2
Ethirimol	0.004	102	100	12.7	5.7	8.1	13.0	22.6
Ethofumesate	0.006	109	98	5.1	5.0	19.8	6.6	30.0
Ethoprophos	0.006	108	98	5.9	5.4	17.1	13.7	31.3
Ethoxyquin	0.007	113	93	12.2	2.1	13.7	9.0	24.1
Ethoxysulfuron	0.003	91	88	3.2	2.5	4.5	17.3	25.5
Etofenprox	0.004	107	102	4.5	6.8	10.3	14.3	25.2
Etoxazole	0.002	98	101	10.7	5.8	6.3	16.2	25.3
Etrimfos	0.007	97	101	10.2	5.4	5.5	6.7	13.7
Famoxadone	0.007	111	100	8.6	3.4	19.7	11.7	33.0
Famphur	0.008	85	90	11.5	8.0	11.2	6.0	19.2
Fenamidone	0.006	112	91	14.0	2.3	17.6	4.4	26.4
Fenamiphos	0.005	78	98	4.8	6.7	4.0	6.2	12.5
Fenamiphos sulfoxide	0.002	84	101	16.5	8.6	10.0	5.7	19.1
Fenamiphos-sulfone	0.009	78	105	7.2	6.0	8.5	4.1	14.8
Fenarimol	0.004	104	105	6.1	3.8	13.8	12.8	27.0
Fenazaquin	0.002	110	101	2.7	3.9	5.5	18.0	26.9
Fenbuconazole	0.005	97	101	14.5	4.2	4.5	11.0	17.8
Fenbutatin Oxide	0.002	95	93	9.1	1.5	6.5	5.5	13.6
Fenchlorphos	0.008	99	95	14.4	9.1	15.5	8.9	27.0
Fenchlorphos-oxon	0.004	89	98	8.4	9.1	11.3	10.2	23.4
Fenhexamid	0.006	100	100	6.3	6.9	8.9	15.0	25.3
Fenobucarb	0.003	111	104	3.7	2.1	19.4	14.6	35.0
Fenoxaprop-P-ethyl	0.006	93	111	7.2	3.6	11.8	10.5	22.9
Fenoxycarb	0.005	99	102	3.4	5.5	7.2	2.3	11.5
Fenpiclonil	0.003	93	110	13.6	4.0	7.8	4.5	14.5
Fenpropathrin	0.003	108	105	5.3	3.3	11.1	4.4	17.3
Fenpropidin	0.003	97	102	11.3	7.1	8.9	11.4	21.2
Fenpropimorph	0.004	97	105	14.1	2.7	9.5	16.6	28.1
Fenpyrazamine	0.002	93	97	7.6	2.5	7.0	1.6	10.8
Fenpyroximate	0.005	94	92	11.3	2.2	10.7	5.0	18.3
Fensulfothion	0.003	101	100	8.9	3.8	12.5	3.5	19.3
Fensulfothion-oxon	0.004	92	102	9.7	3.6	10.9	5.7	18.7
Fensulfothion-oxon sulfone	0.004	93	102	10.9	3.2	18.0	10.7	30.3
Fensulfothion sulfone	0.004	103	112	6.0	4.6	8.3	2.9	14.4

Fenthion	0.007	89	105	13.3	4.5	8.7	4.0	14.6
Fenthion oxon	0.006	95	100	9.8	3.5	4.9	6.8	14.0
Fenthion oxon sulfoxide	0.006	100	101	18.9	5.2	10.5	5.9	19.6
Fenthion sulfone	0.002	104	103	3.3	5.1	15.1	11.6	27.4
Fenthion sulfoxide	0.006	85	97	13.1	6.2	4.2	2.4	8.8
Fentin	0.006	101	93	9.0	7.1	11.7	6.8	21.0
Fentin hidroxide	0.006	91	107	14.6	1.5	6.7	3.2	12.3
Fenvalerate	0.003	86	91	10.4	2.8	10.7	8.3	20.0
Fipronil	0.009	105	104	13.1	5.6	17.3	3.8	26.1
Flazasulfuron	0.006	106	104	8.3	3.5	8.2	3.3	13.5
Flonicamid	0.005	103	103	4.2	3.9	7.4	4.7	13.8
Florasulam	0.006	97	110	19.1	3.4	11.7	4.3	19.8
Florpyrauxifen benzyl	0.002	98	95	5.8	1.5	5.0	3.0	10.0
Fluazifop	0.005	100	101	8.5	3.0	6.3	7.7	14.8
Fluazifop-butyl	0.003	96	95	3.0	2.4	10.6	4.5	16.7
Fluazinam	0.002	95	102	7.6	2.5	3.7	1.7	7.0
Flubendiamide	0.007	116	98	6.2	8.9	16.1	3.7	24.0
Flubenzimine	0.004	110	104	8.0	1.3	14.6	8.5	24.9
Flucarbazone sodium	0.007	84	92	12.0	7.7	5.3	6.5	15.0
Flucycloxuron	0.004	101	91	8.5	1.8	14.0	7.7	22.9
Fludioxonil	0.005	111	100	1.6	3.9	18.9	3.5	27.5
Flufenacet	0.003	105	101	10.3	7.8	10.2	6.6	18.3
Flufenoxuron	0.004	100	103	6.4	4.2	7.7	6.1	15.6
Flufenzine	0.003	90	100	4.5	3.3	11.5	5.1	18.4
Fluometuron	0.006	105	96	5.0	8.7	18.3	5.5	27.5
Fluopicolide	0.007	109	107	11.2	4.4	8.0	2.5	13.4
Fluopyram	0.003	106	93	7.7	2.9	4.8	6.8	12.9
Fluoxastrobin	0.002	91	93	8.3	3.2	15.1	8.7	25.2
Flupyradifurone	0.005	83	92	4.7	3.8	13.1	3.9	19.8
Fluquinconazole	0.004	83	105	8.3	4.1	6.8	4.9	14.9
Flurochloridone	0.006	99	104	9.0	7.7	9.8	5.8	17.6
Fluroxypyr	0.004	95	105	12.1	3.5	6.2	10.9	18.6
Fluroxypyr-1-methylheptylester	0.002	96	102	4.7	5.2	3.6	11.3	17.8
Flusilazole	0.003	86	105	17.0	4.6	7.2	10.0	19.1
Flutolanil	0.006	103	110	7.6	6.5	14.4	14.3	29.3
Flutriafol	0.006	106	97	8.5	4.3	7.9	7.7	16.6

Fluxapyrad	0.004	91	109	17.5	6.1	18.9	8.1	30.1
Fomesafen	0.006	102	90	16.2	4.0	7.1	8.4	17.8
Fonofos	0.003	83	95	11.8	6.6	11.7	3.7	18.2
Foramsulfuron	0.004	92	89	1.2	2.0	14.6	10.6	26.4
Forchlorfenuron	0.003	102	96	4.6	2.8	14.4	2.8	21.3
Formetanate hydrochloride	0.003	106	105	12.0	2.1	5.3	11.3	18.8
Fosthiazate	0.003	105	99	6.0	3.9	10.1	4.2	16.0
Fuberidazole	0.005	81	106	12.7	4.8	18.0	6.8	27.8
Furalaxyl	0.006	95	94	13.8	5.4	15.2	8.1	25.3
Furathiocarb	0.004	92	104	16.3	3.7	18.8	9.2	31.3
Furmecyclox	0.007	98	96	11.2	2.8	10.8	6.8	19.3
Halauxifen free acid	0.004	95	98	12.0	4.7	6.5	4.4	12.5
Halauxifen methyl	0.002	95	102	6.6	1.4	6.7	1.4	10.2
Halfenprox	0.007	99	93	12.9	3.8	10.9	10.7	23.1
Halosulfuron methyl	0.005	108	104	6.8	3.7	16.3	1.3	23.7
Haloxyfop	0.006	105	99	8.0	7.0	15.1	3.6	22.6
Haloxyfop-2-ethoxyethyl	0.006	108	93	7.2	2.5	19.7	6.4	29.9
Haloxyfop-R-methyl	0.003	82	109	7.2	4.3	16.3	9.3	27.4
Heptenophos	0.005	83	111	7.0	3.8	8.5	6.0	15.3
Hexaconazole	0.004	103	101	11.4	4.2	17.8	13.4	32.0
Hexaflumuron	0.005	108	105	6.1	3.2	10.3	7.5	18.5
Hexazinone	0.004	116	102	3.7	7.2	12.3	10.9	23.6
Hexythiazox	0.003	92	104	5.6	6.0	7.5	18.3	28.6
Imazalil	0.005	110	101	3.9	6.1	5.5	5.9	13.8
Imazaquin	0.008	109	102	3.8	2.7	17.7	5.2	27.0
Imazethapyr	0.008	107	94	4.8	2.3	13.3	7.1	22.2
Imibenconazole	0.006	101	100	13.1	2.9	12.9	6.8	22.1
Imidacloprid	0.003	105	99	9.0	6.4	10.9	5.6	18.2
Indoxacarb	0.003	108	106	5.4	4.7	6.2	7.6	15.1
Iodosulfuron-methyl sodium	0.009	97	103	14.3	2.4	15.3	5.0	23.8
Ioxynil	0.005	101	105	5.3	5.3	11.2	4.3	17.7
Ipconazole	0.007	95	94	11.2	2.9	13.9	3.3	21.2
Iprodione	0.006	94	105	13.1	7.0	12.1	7.2	21.4
Iprovalicarb	0.005	86	102	12.4	4.7	3.5	5.3	11.4
Isazofos	0.008	110	100	12.6	2.7	8.0	5.8	15.2
Isophenphos	0.003	101	101	8.6	3.8	14.5	4.7	22.0

Isoprocarb	0.003	82	102	9.9	6.5	7.0	6.2	14.6
Isoproturon	0.004	80	98	10.0	4.4	7.6	10.3	18.7
Isopyrazam	0.006	105	83	8.6	2.4	17.4	6.0	27.0
Isoxadifen-ethyl	0.007	107	90	8.3	5.4	16.9	8.8	27.4
Isoxaflutole	0.008	97	100	19.1	6.5	15.6	7.4	25.6
Isoxathion	0.005	107	90	9.4	5.4	4.5	12.1	19.0
Imazamox	0.007	89	97	11.0	3.3	18.3	2.7	26.7
Imazapyr	0.004	96	92	6.2	1.5	16.7	9.0	27.3
Imizapic	0.005	105	102	5.6	2.7	4.8	2.4	8.5
Indaziflam	0.002	90	96	6.6	3.1	13.9	4.5	21.7
Isoxaben	0.001	87	96	6.9	2.0	17.9	5.9	26.9
Kresoxim methyl	0.006	107	101	7.7	6.5	10.6	8.3	20.0
Lambda-cyhalothrin	0.006	96	95	10.4	4.0	10.6	4.0	17.3
Lenacil	0.005	100	103	15.5	7.8	11.3	16.8	29.9
Linuron	0.003	89	105	18.8	6.3	3.6	3.9	10.8
Lufenuron	0.004	79	95	8.5	5.1	5.1	8.5	15.6
Malaoxon	0.003	88	98	8.1	8.9	3.7	13.4	20.4
Malathion	0.006	101	108	13.4	3.2	12.3	13.0	26.5
Mandipropamid	0.006	101	106	6.3	7.2	12.1	12.0	25.4
MCPA	0.005	105	100	5.7	6.2	6.2	8.0	15.8
MCPA -2-ethylhexyl ester	0.007	87	93	13.4	2.5	10.4	10.1	22.0
MCPA-butyl ester	0.007	86	109	11.1	7.3	9.3	7.2	17.8
MCPA-methyl ester	0.007	96	111	15.5	2.5	19.9	8.4	31.2
MCPB	0.004	107	98	10.0	4.0	16.3	5.7	25.5
Mecarbam	0.004	108	103	8.7	1.1	15.4	13.7	29.6
Mecoprop	0.001	88	90	4.8	1.5	16.0	3.8	24.0
Mefentfluconazole	0.001	91	90	6.2	3.5	5.3	6.2	12.4
Mepanipyrim	0.005	109	86	4.9	2.2	5.6	2.8	10.6
Mephosfolan	0.003	88	98	7.6	1.3	6.4	6.3	13.4
Mepronil	0.004	110	99	5.5	5.4	17.0	12.7	30.4
Meptydinocap	0.005	84	100	10.3	5.2	6.8	7.5	16.4
Mesosulfuron methyl	0.003	88	91	10.0	1.8	6.7	3.8	12.0
Mesotrione	0.006	112	110	4.1	4.2	5.0	7.7	14.0
Metaflumizone	0.007	88	94	12.9	4.4	9.6	9.9	20.3
Metalaxyl/ Metalaxyl M	0.003	95	99	3.8	5.5	3.8	11.6	17.6
Metamitron	0.005	96	104	10.4	5.9	9.8	9.7	20.1

Metazachlor	0.003	101	104	6.5	4.9	17.4	9.1	28.2
Metconazole	0.004	88	104	16.0	6.0	4.9	11.2	18.6
Methabenzthiazuron	0.005	107	100	12.9	5.8	11.6	10.9	24.1
Methacrifos	0.003	90	98	15.6	7.1	13.5	9.3	24.8
Methamidophos	0.003	88	101	9.9	6.2	5.4	4.6	11.3
Methidathion	0.004	78	101	1.1	6.4	11.2	2.9	17.1
Methiocarb	0.004	101	102	4.0	6.2	17.8	14.8	33.1
Methiocarb sulfone	0.005	80	100	9.5	7.6	8.3	9.4	18.4
Methiocarb sulfoxide	0.003	97	104	5.5	4.4	4.9	13.2	20.6
Methomyl	0.004	91	104	18.3	2.6	9.7	14.3	26.5
Methoprene	0.009	96	96	15.0	3.0	12.6	7.7	21.9
Methoprotryne	0.007	96	101	17.1	4.7	8.4	8.1	17.9
Methoxyfenozide	0.005	108	104	5.0	7.9	15.2	12.9	28.7
Metobromuron	0.006	101	102	15.4	4.3	6.7	7.5	16.1
Metolachlor	0.005	111	100	7.9	5.8	17.0	8.0	27.0
Metolcarb	0.006	110	104	6.9	5.2	13.5	9.2	23.9
Metosulam	0.005	116	104	2.2	5.1	17.9	10.7	29.7
Metoxuron	0.003	98	102	10.9	5.2	9.5	10.1	20.3
Metrafenone	0.005	91	104	14.4	2.4	9.6	11.1	21.7
Metribuzin	0.005	108	102	6.8	4.7	14.3	11.4	26.3
Metsulfuron-methyl	0.005	101	110	15.3	1.3	16.2	9.7	27.6
Mevinphos	0.004	103	106	9.9	2.3	6.6	8.3	15.8
Milbectin A3	0.004	83	93	7.6	2.8	5.7	3.2	11.1
Milbectin A4	0.003	104	97	8.5	4.3	19.6	7.0	30.5
Molinate	0.007	97	107	15.4	3.4	13.4	6.1	22.0
Monocrotophos	0.006	92	95	3.1	2.8	11.6	7.2	19.9
Monolinuron	0.004	104	98	10.7	5.8	12.9	10.0	23.8
Monuron	0.004	108	113	15.5	1.8	8.2	1.1	13.6
Myclobutanil	0.004	110	109	11.0	3.1	14.5	15.9	31.5
N,N-dimethylformamide	0.004	92	105	12.4	2.8	9.5	8.1	19.4
N-2,4-dimethylphenyl-N'-methylformamidine	0.006	106	106	5.5	6.9	8.5	7.3	16.3
Napropamide	0.008	99	103	13.3	6.1	19.2	4.5	28.5
Neburon	0.005	109	102	7.9	3.7	13.1	6.2	21.1
Nicosulfuron	0.003	79	95	10.4	2.0	19.6	3.7	28.8
Nitenpyram	0.002	101	103	12.5	5.1	6.6	15.9	25.6
Nitrapyrin	0.003	98	98	11.2	7.1	7.9	8.4	18.2

Norflurazon	0.005	95	96	16.4	2.8	16.6	4.6	25.3
Novaluron	0.004	89	91	13.7	1.8	17.7	3.5	26.4
Nuarimol	0.006	99	106	4.8	7.2	5.9	4.3	12.0
Ofurace	0.004	99	100	13.4	1.5	4.4	8.8	15.1
Omethoate	0.006	86	103	11.1	3.8	6.0	4.1	12.2
Orthosulfamuron	0.005	81	91	7.4	2.9	11.6	14.2	26.4
Oxadixyl	0.004	101	106	6.1	4.1	15.9	8.5	25.7
Oxamyl	0.002	109	105	10.9	6.0	7.0	16.4	25.8
Oxasulfuron	0.005	86	96	11.4	2.6	5.5	3.2	10.1
Oxycarboxin	0.007	111	104	2.1	1.3	10.8	4.3	16.8
Paclobutrazol	0.003	104	96	3.8	3.3	15.7	9.4	26.3
Paraoxon-ethyl	0.005	101	101	10.1	5.8	7.3	3.4	13.7
Paraoxon-methyl	0.004	101	100	6.1	8.9	4.4	12.2	19.0
Pebulate	0.008	96	101	11.5	2.8	18.7	9.4	30.4
Penconazole	0.005	108	98	10.5	5.1	14.6	11.4	26.9
Pencycuron	0.003	113	106	4.4	6.0	18.0	13.7	32.3
Pendimethalin	0.003	101	106	11.7	3.8	8.8	13.3	23.2
Penflufen	0.002	91	96	5.9	1.5	8.2	3.0	13.1
Penoxsulam	0.004	99	91	7.0	2.0	9.4	10.5	20.3
Penthiopyrad	0.003	89	99	7.1	1.6	11.2	2.9	17.5
Phenmedipham	0.005	79	110	7.1	3.2	7.1	3.9	12.3
Phenothrin	0.007	96	89	12.1	2.3	14.2	6.2	23.7
Phenthoate	0.005	112	100	4.7	8.0	4.5	11.3	18.0
Phosalone	0.002	102	105	4.8	6.2	14.5	5.7	22.5
Phosmet	0.005	117	107	2.7	3.9	19.7	10.1	31.6
Phosmet-oxon	0.008	102	91	10.0	2.6	14.0	4.5	21.5
Phosphamidon	0.003	108	107	5.3	4.3	7.3	12.2	20.6
Phoxim	0.005	97	101	11.1	1.7	12.1	8.4	21.7
Picloram	0.008	93	107	16.8	3.4	12.5	2.2	19.4
Picolinafen	0.006	97	107	12.1	0.7	19.5	10.6	32.0
Picoxystrobin	0.005	101	90	11.2	1.4	19.7	6.7	29.9
Pinoxaden	0.004	77	92	6.1	2.6	8.1	5.6	14.3
Pirimicarb	0.003	105	106	5.8	7.8	5.1	6.6	12.6
Pirimicarb-desmethyl	0.002	105	111	6.6	1.7	5.2	6.4	12.1
Pirimiphos-methyl	0.002	95	105	5.1	9.2	7.9	10.7	19.4
Pirimiphos-ethyl	0.006	96	91	16.1	2.3	13.5	4.6	21.2

Prochloraz	0.005	105	106	3.3	7.0	15.6	8.9	26.0
Profenofos	0.003	105	105	3.1	6.1	8.7	17.0	27.3
Profoxidim	0.003	101	89	11.9	1.9	17.1	8.0	27.9
Prohexadione calcium	0.007	85	90	17.8	2.5	4.6	5.5	12.4
Prometryn	0.003	106	110	7.8	2.4	9.2	10.4	20.2
Propachlor	0.006	98	106	18.4	7.9	8.8	7.2	18.2
Propamocarb	0.003	100	110	4.1	2.4	4.9	9.5	15.6
Propamocarb-hydrochloride	0.005	84	104	12.4	2.2	18.2	4.5	27.0
Propanil	0.005	92	105	10.2	3.2	9.4	4.1	16.1
Propaquizafop	0.007	101	100	11.2	2.2	10.7	7.9	19.4
Propargite	0.003	115	104	2.6	6.6	12.1	10.6	23.3
Propazine	0.004	88	102	17.7	6.8	3.3	7.3	14.0
Propetamphos	0.005	90	108	14.6	6.6	8.6	2.9	14.6
Propham	0.004	86	105	8.2	3.4	19.0	7.5	29.8
Propiconazole	0.003	109	105	6.0	3.7	7.8	13.2	22.3
Propoxur	0.004	109	108	2.0	4.3	11.5	4.3	18.1
Propoxycarbazone-sodium	0.006	88	93	10.7	3.9	10.1	4.1	16.5
Propyzamide	0.005	103	105	12.9	6.0	3.5	7.9	15.0
Proquinazid	0.005	89	99	11.5	1.9	10.6	10.0	21.1
Prosulfocarb	0.006	98	93	10.4	0.9	13.9	7.9	23.2
Prosulfuron	0.004	82	109	13.3	5.9	12.3	7.4	21.3
Prothioconazole	0.003	82	104	11.5	8.1	5.6	3.8	12.1
Prothioconazole desthio	0.002	92	93	3.7	2.1	18.3	2.3	26.7
Prothiophos	0.004	111	110	3.8	1.6	18.2	13.2	31.9
Pymetrozine	0.003	107	107	5.4	1.9	17.8	6.8	27.4
Pyraclostrobin	0.005	95	106	13.8	5.6	9.2	15.3	26.0
Pyraflufen-ethyl	0.005	94	103	18.1	7.9	10.5	14.9	27.0
Pyrazophos	0.002	113	104	3.8	2.8	17.3	11.5	29.6
Pyretrins	0.004	102	93	11.0	1.9	14.6	1.6	21.6
Pyridaben	0.003	113	108	3.0	5.4	16.2	6.8	25.1
Pyridalyl	0.004	94	109	5.7	6.0	18.1	10.2	29.6
Pyridaphenthion	0.005	105	102	3.9	4.9	13.6	11.1	25.2
Pyridate	0.001	90	94	15.6	1.9	17.8	7.8	28.2
Pyrifenox	0.006	90	102	14.7	7.7	5.6	11.2	19.0
Pyrimethanil	0.003	99	101	16.0	4.2	15.3	7.1	24.9
Pyrimidifen	0.002	91	98	6.6	1.9	7.3	10.3	18.2

Pyriofenone	0.003	92	98	5.8	1.8	6.0	5.2	11.9
Pyriproxyfen	0.002	108	106	5.4	5.9	15.6	11.0	27.2
Pyroxasulfone	0.003	96	102	5.6	5.5	9.8	6.5	17.8
Pyroxsulam	0.004	103	103	5.4	4.0	5.8	5.1	13.5
Quinalphos	0.007	105	102	11.2	7.4	10.8	4.1	17.4
Quinclorac	0.004	94	90	15.8	3.7	19.0	8.1	30.2
Quinmerac	0.002	90	87	7.9	1.6	19.9	12.6	33.9
Quinomethionate	0.006	93	101	19.4	4.2	15.0	6.8	25.0
Quinoxyfen	0.002	101	106	4.9	4.7	6.8	11.8	19.7
Quizalofop ethyl	0.004	86	104	11.8	3.6	8.0	17.6	28.0
Resmethrin	0.003	104	103	2.6	5.2	12.3	11.8	24.6
Rimsulfuron	0.004	108	103	6.5	5.1	8.2	9.5	18.5
Sedaxane	0.003	91	107	10.2	3.4	6.6	5.4	14.1
Sethoxydim	0.004	107	88	5.8	2.9	11.6	5.7	18.8
Silthiofam	0.007	92	95	7.7	2.5	11.0	5.1	18.0
Simazine	0.004	91	108	17.7	4.9	5.0	5.1	13.5
S-Metolachlor	0.007	108	89	5.8	3.8	9.4	5.2	15.6
Spinetoram	0.006	106	90	8.4	2.9	15.9	1.4	23.1
Spinosad	0.004	100	109	6.9	7.5	18.3	11.8	31.5
Spirodiclofen	0.002	92	91	2.3	0.9	14.8	7.1	23.4
Spiromesifen	0.005	79	94	13.9	3.1	11.8	9.1	21.8
Spirotetramat	0.003	96	108	15.9	2.7	14.3	6.4	23.0
Spirotetramat-enol-glucoside	0.003	91	90	4.8	2.2	8.6	5.2	15.8
Spirotetramat-mono-hydroxy	0.005	91	97	5.4	3.0	7.2	5.0	14.1
Spirotetramat-enol	0.005	104	100	8.6	5.2	16.7	6.8	26.8
Spirotetramat-keto	0.003	90	92	5.5	2.0	10.3	10.6	21.6
Spiroxamine	0.003	104	105	6.1	4.9	6.0	8.2	14.8
Sulfosulfuron	0.007	107	99	13.2	3.3	8.2	3.8	15.3
Sulfotep	0.005	92	89	16.6	3.0	12.8	7.9	22.1
Sulfoxaflor	0.003	96	102	9.0	1.9	18.6	13.9	33.1
Tau-fluvalinate	0.005	100	99	5.9	3.7	16.2	4.1	25.0
Tebuconazole	0.006	113	103	4.3	5.3	11.8	12.0	24.4
Tebufenozide	0.003	106	106	7.2	9.1	13.2	14.6	28.7
Tebufenpyrad	0.002	100	105	3.0	4.5	6.2	10.7	17.9
Tebutam	0.005	106	101	9.0	7.5	7.3	12.5	21.5
Teflubenzuron	0.005	104	92	5.1	4.2	8.3	7.6	16.5

Tembotrione	0.005	77	105	8.8	1.8	10.2	6.7	19.2
Tembotrione metabolite	0.006	79	107	6.4	2.4	7.2	6.2	14.7
Tepraloxydim	0.006	96	101	9.7	5.6	11.8	9.3	22.1
Terbacil	0.005	94	93	11.6	10.6	7.4	6.4	16.1
Terbufos	0.003	111	106	3.5	5.7	19.5	12.5	33.0
Terbumeton	0.008	100	94	13.0	3.0	18.9	10.1	30.8
Terbuthylazine	0.003	90	108	10.3	3.7	6.6	9.0	16.8
Terbuthylazine-desethyl	0.003	112	106	5.8	6.8	16.5	8.8	27.2
Terbutryn	0.002	113	110	2.9	4.9	13.1	11.0	24.4
Tetrachlorvinphos	0.006	81	108	11.2	2.9	4.7	5.0	11.2
Tetraconazole	0.004	102	104	10.4	7.5	13.8	12.4	27.3
Tetramethrin	0.001	91	93	5.0	2.2	4.2	1.6	7.5
Thiabendazole	0.002	113	108	7.3	1.9	9.8	2.5	15.4
Thiacloprid	0.003	110	106	4.4	6.0	12.5	6.9	20.7
Thiamethoxam	0.005	102	109	7.1	6.1	16.2	9.4	26.9
Thidiazuron	0.004	94	107	15.9	7.8	15.6	9.7	27.1
Thiencarbazone-methyl	0.006	96	93	7.8	3.3	9.7	5.2	18.7
Thifensulfuron-methyl	0.006	106	91	10.3	2.3	12.5	9.6	23.2
Thiobencarb	0.007	105	98	9.7	2.5	9.0	4.8	16.1
Thiodicarb	0.005	110	102	6.6	5.4	18.9	10.9	31.1
Thiofanox-sulfoxide	0.005	99	107	13.0	5.1	17.8	10.6	30.3
Thiometon	0.007	88	104	7.9	5.2	13.3	3.9	20.8
Thiophonate-methyl	0.004	104	100	9.4	7.0	6.7	4.8	13.0
Thiram	0.002	91	100	8.2	1.5	8.4	7.1	15.9
Tolclophos-methyl	0.002	105	108	7.3	3.9	7.5	7.8	16.3
Tolyfenpyrad	0.002	91	92	5.2	1.9	7.1	2.8	11.4
Tolyfluanide	0.003	102	106	15.3	5.0	12.8	14.2	27.8
Tralkoxydim	0.006	107	90	7.8	2.2	9.1	5.5	15.5
Triadimefon	0.003	104	106	12.4	4.6	10.2	6.8	18.4
Triadimenol	0.006	106	103	9.1	4.9	19.2	1.8	28.1
Tri-allate	0.003	89	96	13.5	2.6	7.9	4.2	13.7
Triasulfuron	0.006	104	103	12.8	5.0	10.9	5.6	18.6
Triazamate	0.007	100	103	8.7	1.9	4.0	4.3	10.5
Triazophos	0.006	107	106	6.5	4.9	13.3	16.3	30.0
Tribenuron-methyl	0.003	98	104	17.5	8.3	6.7	5.4	14.3
Trichlorfon	0.004	96	104	12.1	5.8	7.2	4.8	14.4

Triclopyr	0.006	84	87	10.9	3.0	17.0	4.4	25.7
Tricyclazole	0.008	101	105	11.7	4.3	12.7	4.9	20.3
Tridemorph	0.008	101	85	10.9	2.8	11.2	6.3	19.1
Trifloxystrobin	0.002	110	106	1.4	5.7	18.6	11.4	31.0
Trifloxysulfuron-sodium	0.003	96	99	11.5	7.0	7.1	12.9	23.3
Triflumizole	0.004	107	106	8.2	5.8	14.6	5.4	22.6
Triflumuron	0.004	102	106	8.7	5.4	15.2	4.6	23.0
Triflusulfuron-methyl	0.002	83	88	4.2	4.3	4.2	7.8	13.6
Triforine	0.005	96	89	3.0	3.0	12.4	7.0	20.6
Trinexapac	0.007	96	94	3.0	6.1	16.1	5.4	24.6
Triticonazole	0.003	105	111	4.8	5.2	15.1	8.7	25.1
Tritosulfuron	0.008	102	87	13.9	6.1	9.3	5.4	20.0
Uniconazole	0.007	104	101	5.2	2.1	7.0	7.2	15.0
Valifenalate	0.002	97	96	5.6	2.0	14.1	2.3	21.4
Vamidothion	0.008	90	104	16.2	3.3	10.5	4.8	17.9
Zoxamide	0.005	102	105	16.8	7.7	6.7	12.6	21.5
